# Metastatic Bladder Cancer: A Review of Current Management

**DOI:** 10.5402/2011/545241

**Published:** 2011-06-09

**Authors:** Andrew Fletcher, Ananya Choudhury, Nooreen Alam

**Affiliations:** ^1^Department of Palliative and Supportive Care, The Christie NHS Foundation Trust, Wilmslow Road, Manchester M20 4BX, UK; ^2^Department of Clinical Oncology, The Christie NHS Foundation Trust, Wilmslow Road, Manchester M20 4BX, UK; ^3^The University of Manchester, Oxford Road, Manchester, M13 9PL, UK

## Abstract

Bladder cancer continues to result in substantial morbidity and mortality for affected individuals. Advances in the management of metastatic bladder cancer have been limited. Chemotherapy with platinum-based regimes remains the mainstay of first-line treatment. Studies investigating alternative regimes have offered no survival advantage. Targeted therapies may offer benefit either as single agent or in combination with chemotherapy. Symptoms due to metastatic bladder cancer impact patients' quality of life, and therefore holistic management is vital. Such management includes radiotherapy, bisphosphonates, and the involvement of specialist palliative care services. This review will discuss the current management for metastatic bladder cancer, future potential treatment modalities, and the evidence to support the management strategies.

## 1. Introduction

There were over 10,000 new cases of bladder cancer in the UK in 2008 making it the 7th commonest cancer overall. A quarter of these are muscle-invasive bladder cancers (MIBC) [1–5]. Approximately 5% of patients present with metastatic bladder cancer at diagnosis [5]. In the UK, the most common histological type of bladder cancer is transitional cell carcinoma (TCC), however, there are other types, including squamous cell carcinoma, adenocarcinoma, and less commonly small cell and small cell and sarcoma. Factors such as geography and underlying aetiology influence prevalence across the world [6]. This review will discuss the current management of patients with metastatic bladder cancer, in the main of the TCC type, and the evidence to support this management.

## 2. Chemotherapy

### 2.1. First-Line Chemotherapy

Combination chemotherapy is the treatment of choice for metastatic bladder cancer. Methotrexate, vinblastine, doxorubicin, and cisplatin (MVAC) was for many years the preferred regime; however, patients experienced high toxicity levels. Newer chemotherapy regimes have attempted to offer comparable or better efficacy in terms of overall survival, response rates, and time to disease progression whilst decreasing toxicity (Table 1).

In an attempt to offer the benefits of MVAC whilst reducing toxicity, especially neutropenic sepsis, Sternberg et al. [7] undertook a multicentre study of 263 patients with metastatic or advanced urothelial tumours who had not previously received systemic chemotherapy and randomised them to receive traditional MVAC or high-dose-intensity MVAC plus granulocyte colony-stimulating factor (HD-MVAC + GCSF). HD-MVAC + GCSF when compared with MVAC offered statistically lower levels of grade 3 and 4 neutropenia (20% versus 62%, *P* < .001). Although median survival was unaffected, there was a statistically significant improvement in complete response (25% versus 11%, *P* = .006) and overall response rate (70% versus 58%, *P* = .016).

Gemcitabine and cisplatin (GC) been compared with MVAC in a phase-III randomised controlled trial, which showed that GC had significantly less toxicity with significantly lower rates of neutropenic sepsis and grade 3 or 4 mucositis and a reduction in drug-related mortality, though the latter was not statistically significant [8]. Response rates for GC versus MVAC were 49.4% and 45.7%, respectively, median survival of 13.8 and 14.8 months, and the time to progressive disease was identical in both groups at 7.4 months. Furthermore, there were statistically significant higher number of patients with a greater than 5% increase in weight in the GC group and a reduction in fatigue, although this was not significant. Other quality-of-life markers were maintained and were similar in both arms.

Triplet combinations with GC have also been studied, including a single-centre study of the triplet combination of GC plus paclitaxel [9]. Median survival and median progression-free survival were 18.5 months and 7 months, respectively, with a reported response rate of 64.7%. Neutropenia was experienced in 41.2% of participants and neutropenic sepsis in 32.4%.

Platinum-containing chemotherapy is the gold standard for patients with metastatic bladder cancer, however, some patients have inadequate renal function or do not tolerate cisplatin, for example due to neuropathy. In these patients, carboplatin has been suggested as an alternative. Bellmunt et al. [10], Dogliotti et al. [11], and Dreicer et al. [12] compared cisplatin with carboplatin-containing regimes. Bellmunt et al. [10] randomised 47 patients to MVAC or methotrexate, carboplatin, and vinblastine (M-CAVI). There was a statistically higher median survival in the MVAC group (16 months versus 9 months, *P* = .03); however, response rates were similar. Gemcitabine plus carboplatin was compared with GC in a study of 110 chemonaive patients by Dogliotti et al. [11]. No statistically significant differences were demonstrated in terms of response rate (40% versus 49.1%), complete response (1.8% versus 14.5%), median time to progression (7.7 months versus 8.3 months), median survival (9.8 months versus 12.8 months), or toxicity. Dreicer et al. [12] undertook a phase-III study comparing MVAC versus paclitaxel plus carboplatin, following observation of previous good response. In this trial, there was no statistically significant improvement in overall survival and response rates or difference in quality of life, but there were higher rates of neutropenia in those receiving MVAC. 

A further study by Carles et al. [13] undertook a multicentre phase-II study using oxaliplatin and gemcitabine. Chemo-naïve as well as patients who had received previous chemotherapy were permitted to enter the study. Complete response was 6.5% with an overall response rate of 48%. At the end of study, most of the patients (90%) had progressive disease with a median time to disease progression of 5 months and a median overall survival of 6.5 months. Grade 3 or 4 neutropenia was reported in 22% of cases.

### 2.2. Second-Line Chemotherapy

Potential second-line options in metastatic bladder cancer include single-agent vinflunine, taxanes and combination regimes (Table 2). Studies often select patients with performance status 0 and 1 for their inclusion criteria, which may not reflect the generally poor-performance status represented by patients with metastatic bladder cancer.

A phase-III multi-centre study randomised 370 patients who had previously progressed through platinum-based chemotherapy, to receive vinflunine and best supportive care versus best supportive care alone [14]. Overall survival and progression-free survival were marginally, albeit significantly longer in those receiving vinflunine, 6.9 months versus 4.3 months (*P* = .04), and 3 months versus 1.5 months, respectively. Vinflunine did not impair quality of life. 

A phase-II study recruited 45 patients who were treated with weekly paclitaxel [15]. Overall response was 9%, complete response 2%, median time to progression 3 months, and median survival 7 months. Febrile neutropenia rates were 4%. Two weekly versus 3 weekly gemcitabine plus paclitaxel was investigated by Fechner et al. [16]. Median time to progression and median survival were not statistically different, however, there was a statistically significant increase in complete response rate in the 3-weekly regime, though this was a small study.

Pemetrexed with vitamin B12, folic acid, and dexamethasone prophylaxis was investigated in a phase-II study to treat 47 patients with progressive disease following adjuvant or neoadjuvant chemotherapy [17]. Complete response, partial response, and overall response were 6.4%, 21.3%, and 27.7%, respectively. Median duration of response was 5 months and median overall survival 9.6 months. Neutropenic rates were 4.3%.

## 3. Targeted Therapies

### 3.1. Histone Deacetylase Inhibitors (HDACi)

An imbalance in the equilibrium of histone acetylation has been associated with carcinogenesis and cancer progression. HDAC inhibitors (HDACis) lead to an accumulation of acetylated histone proteins in both tumour cells and in normal tissues. They activate differentiation, arrest the cell cycle in G1 and/or G2, and induce apoptosis in transformed or cancer cells. In vitro and in vivo, HDACi demonstrate antitumour activity, by leading to an accumulation of acetylated histones and cell cycle arrest and/or apoptosis of some transformed cells.

HDACi are currently being investigated in ongoing research studies both in the locally advanced and metastatic setting [23]. Early phase trials have also investigated using HDACi concurrently with radiotherapy and chemoradiotherapy [24–27].

### 3.2. Epidermal Growth-Factor-Receptor-Targeted Therapy

Overexpression of epidermal growth factor receptors including human epidermal growth factor receptor (EGFR/HER) has been demonstrated in bladder cancer and may offer both prognostic indicators as well as a potential targeted treatment for metastatic bladder cancer with recommendations suggesting to be used as a supplement to conventional chemotherapy.

#### 3.2.1. Trastuzumab

Trials are currently ongoing; however, a small study by Peyromaure et al. [28] included 6 patients with metastatic urothelial cancer and overexpression of HER-2. The participants received trastuzumab and paclitaxel; carboplatin, trastuzumab, and paclitaxel, or trastuzumab alone. After 3 cycles, all patients demonstrated a partial response between 30 and 70%. After 6 cycles, 5 patients had progression of at least 1 site of metastatic disease. In the five patients who died, the interval between trastuzumab initiation and patient death ranged from 8 to 18 months. One patient who received trastuzumab alone remained alive at 28 months. Grade 4 neutropenia was documented in 1 patient, and there were no cases of congestive heart failure. The small number of patients included in the study limit the conclusions that can be drawn from the results, however, highlight the need for larger trials to investigate the effectiveness of trastuzumab therapy alone and in combination with chemotherapy. 

The safety and efficacy of trastuzumab in combination with carboplatin, gemcitabine, and paclitaxel was investigated as part of a phase-II National Cancer Institute trial [18]. Patients with over expression of HER-2/neu receptor on immunohistochemistry, gene amplification, and/or elevated serum HER-2/neu, who had not received chemotherapy for metastatic disease, were treated with a regime comprising all the four drugs. In those treated, there was a 70% overall response with a complete response in 11.4%. Median time to progression was 9.3 months and median survival 14 months. Toxicities included: grade 3 neutropenia (16%), grade 4 neutropenia (70%), infection-related deaths (5%), overall cardiac toxicity (14%), and grade III sensory neuropathy (14%). 

In a further phase-II study, Beuzeboc et al. [29] aimed to demonstrate a progression-free survival benefit for those treated with trastuzumab. Interim safety results concluded that there were no statistically significant differences in the rates of toxicity between those receiving trastuzumab plus chemotherapy versus chemotherapy alone. Efficacy data is awaited.

#### 3.2.2. Lapatinib

Lapatinib has an established use in HER-2-positive breast cancer [30] and is being increasingly trailed in bladder cancer. In a single-arm phase-II study using Lapatinib as the second-line treatment, there is a correlation between overexpression of eGFR/HER-2 and response to this therapy; however, the statistically significant benefit relates to the overexpression of eGFR [19].

### 3.3. Vascular Endothelial-Growth-Factor-Targeted Therapy

A study by Bochner et al. [31] explored the association between traditional prognostic indicators and tumour angiogenesis. Estimated probability of recurrence at 5 years for lowest microvessel counts compared with highest counts were 19% and 68%; respectively, and overall 5 year survival was 68% compared to 34%. Tumour angiogenesis is an independent prognostic indicator in patients with invasive bladder TCC. Therapies that block vascular endothelial growth factor and thus the development of microvessels may offer an alternative treatment in patients with metastatic bladder cancer. Current phase-II studies investigating the use of bevacizumab have shown encouraging results; however, there are concerns with regards to toxicity. One such study which combined bevacizumab with cisplatin and gemcitabine as first-line chemotherapy identified complete response in 17% of patients with an overall response rate of 67% [20]. Although the median time to progression appeared comparable to earlier studies using chemotherapy alone, the median survival appeared to be higher at 19.1 months. There were three treatment-related deaths, and 21% of patients developed venous thromboembolism.

### 3.4. Proteosome Inhibitors

Inhibitors of the 26S proteaosome complex have been shown to arrest tumour spread and growth and suppress angiogenesis; therefore, it is suggested that they may offer potential therapeutic approaches. Bortezomib, a proteosome inhibitor, has been investigated in two phase-II single-arm studies to treat advanced or metastatic urothelial cancers [21, 22]. Both concluded that single-agent Bortezomib is ineffective and does not offer antitumour activity in this patient group.

## 4. Radiotherapy

Radiotherapy has several roles in the management of metastatic bladder cancer including palliation of pain secondary to bone-metastases, control of advancing pelvic pathology, and a reduction in urinary symptoms, for example haematuria. A variety of potential regimes have been investigated for the efficacy and toxicity. 

An MRC BA09 study by Duchesne et al. [32] compared two hypofractionated radiotherapy schedules for local symptom control of muscle-invasive bladder cancer, 35 Gy in 10 fractions over two weeks versus 21 Gy in 3 fractions over one week. 500 patients were initially randomised with 3-month follow-up data available in 272 patients. Overall symptom improvement, defined as improvement of at least 1 symptom by 1 grade without worsening another symptom, was 71% in those receiving 35-Gy compared with 64% in the 21-Gy arm, though this was not statistically significant. Comparing the 35 Gy group with the 21 Gy group for patients with specific pretreatment symptoms, urinary frequency resolved in 43% and 42%, respectively, nocturia in 51% and 35%, haematuria in 58% and 61%, and dysuria in 47% and 49%. No difference in survival or change in WHO performance status was demonstrated between the two treatment arms. Two-thirds of participants reported that quality-of-life symptom scores were either unchanged or improved three months after treatment. A smaller study by McLaran et al. [33] treated 65 elderly patients with muscle-invasive bladder cancer. Treatment involved 30 Gy in 5 fractions on a weekly basis with an additional 6 Gy given as the sixth fraction for participants who were fit, tolerated previous treatment, and had a field size <1000 cm^3^. Complete palliation of symptoms was achieved in 51% of participants. Frequency and dysuria resolved in 24% and haematuria resolved in 92% and frequency and dysuria resolving in 24%. Twenty-eight patients experienced transient worsening of their urinary symptoms with eight requiring hospital admission due to toxicities. The median symptom-free interval was 7 months (range 0–40 months). 

 Other common fractionation regimes in use are 20 Gy in 5 fractions over one week or a single 8–10 Gy fraction.

Several large studies investigating the use of radiotherapy for painful bone-metastases have been reported, comparing single with multiple fraction regimes. A study from the Bone Pain Trial working party [34] compared 8-Gy single-fraction radiotherapy with a multifraction regime (20 Gy in 5 fractions or 30 Gy in 10 fractions) for the treatment of skeletal-related pain in 765 patients. There were no differences demonstrated in the time to response or to first increase in pain at any time up to 12 months. Despite retreatment being twice as common in those treated with a single fraction, the authors concluded that retreatment reflected a greater readiness to retreat after a single fraction, rather than a greater need. A further study by Steenland et al. [35] compared 8 Gy single-fraction radiotherapy with 24 Gy in 6 fractions in 1171 patients with metastatic cancer to bone. There were no statistical differences between the two treatment arms in terms of response, change in pain medication requirements, quality of life, or side effects. Retreatment rates were higher in those receiving a single fraction. Although level of pain was the main reason for retreatment, the authors concluded, as they did in the previous study discussed, that after a single fraction of radiotherapy, doctors were more willing to retreat. Other studies comparing single- with multifraction radiotherapy for the treatment of bone-metastases-related pain have shown comparable results [36, 37]. A Cochrane review drew similar conclusions to the evidence discussed concluding that single-fraction and multifraction radiotherapy were equally effective in relieving pain, however, single-fraction radiotherapy conferred an increased need for retreatment and higher risk of pathological fractures [38]. It was also stated that quality-of-life and economic data is required to identify the optimal treatment regime.

## 5. Bisphosphonates

Bisphosphonates, as with other cancers, have essentially three roles: the management of hypercalcaemia of malignancy, reduction in skeletal-related events in patients with bone-metastases and the management of pain related to bone-metastases. It is widely accepted that bisphosphonates reduce the risk of skeletal complications in multiple cancers, especially metastatic disease from solid tumours [39–42] and myeloma [43], and can assist the management of pain due to bone-metastases. Although more limited, there is literature to support this theory in patients with bone-metastases from bladder cancer. A study by Zaghoul et al. [42] evaluated the effect of zoledronic acid on skeletal-related events. Patients receiving palliative radiotherapy were randomised to placebo or zoledronic acid for 6 months. They concluded that the patients receiving zoledronic acid had a lower incidence of skeletal-related events, a prolonged time to the first skeletal-related event and an increased 1-year survival. 

The recommended dose to prevent skeletal-related events in patients with bone-metastases is 4 mg zoledronic acid (Zometa), with the dose adjusted based on baseline creatinine clearance (CrCl) in mL/minute [44].

## 6. Palliative Care

All the treatment options discussed are being given with palliative intent to achieve a degree of disease control and aid symptom management. Improving Outcomes in Urological Cancers published by the National Institute of Clinical Excellence in September 2002 stated that palliative care is an integral part of the management of patients with urological cancers and should be available if needed to provide symptom control as well as social, spiritual, and psychological support [45]. 

This belief is supported by Brierly and O'Brien [46] who reviewed urology outpatient attendances as well as inpatient admissions and concluded that palliative care services should be closely involved in the management of patients with urological cancers and that urologists should develop the skills required to offer palliative care to their patients. 75% of patients with terminal malignancy attending outpatients had problems that would have benefited from specialist palliative care; however, the number may be higher as psychosocial aspects were not addressed during the assessments [46].

## 7. Conclusions

First-line chemotherapy in the metastatic setting is GC or HD-MVAC + GCSF. The regimes offer comparable effectiveness with reduced toxicity levels compared with the traditional MVAC regime. Carboplatin-based chemotherapy regimes offer an alternative to cisplatin-based regimes where cisplatin is contraindicated. Literature reflecting second-line chemotherapy is limited and needs further investigations. The novel targeted therapies discussed may offer additional therapeutic options either in combination with chemotherapy or as single agents. Further studies would also benefit from a greater emphasis on quality-of-life measures including pain control, which in the palliative setting, is as important as the potential response to treatment or survival advantage.

## 8. Key Points

- First-line chemotherapy in the metastatic setting is gemcitabine plus cisplatin or high dose intensity methotrexate, vinblastine, doxorubicin, and cisplatin (MVAC) plus granulocyte-colony-stimulating factor (GCSF).
- Novel targeted therapies offer potential additional therapeutic options; however, additional research is required.
- Radiotherapy either single- or multifraction regimes offer symptomatic benefits for bone pain and localised pelvic symptoms.
- Patients with metastatic urothelial cancer demonstrate substantial morbidity that would benefit from close involvement with palliative care services.

## Figures and Tables

**Table 1:** Summary of literature related to first-line chemotherapy for metastatic bladder cancer.

	Sternberg et al. [7]	Sternberg et al. [7]	Von der Masse et al. [8]	Von der Masse et al. [8]	Ecke et al. [9]	Bellmunt et al. [10]	Bellmunt et al. [10]	Dogliotti et al. [11]	Dogliotti et al. [11]	Dreicer et al. [12]	Dreicer et al. [12]
Chemotherapy regime	MVAC	HD-MVAC + GCSF	MVAC	GC	GC + Pac	MVAC	M-CAVI	GC	Gem-Carbo	MVAC	Carbo-Pac
Response Rate (%)	58	72	45.7	49.4	64.7	52	39	49.1	40	35.9	28.2
Complete Response rate (%)	11	25	11.9	12.2	29.4	12.5	0	14.5	1.8	12.8	2.6
Median time to progressive disease (months)	9.6	11.1	7.4	7.4	7			8.3	7.7	8.7	5.2
Median survival (months)	14.1	15.5	14.8	13.8	18.5	16	9	12.8	9.8	15.4	13.8
Toxicity											
Grade 3 or 4 Neutropenia (%)	62	20	82	71	41.2			34.6	45.4	77	29
Neutropenic sepsis or febrile neutropenia (%)	26	10	12	1	32.4	18	3.2				
Drug-related deaths (%)	4	3	3	1		4	0			2.3	2.4

**Table 2:** Summary of literature related to second-line chemotherapy and targeted therapies for metastatic bladder cancer.

	Chemotherapy						Targeted Therapies				
	Bellmunt et al. [14]	Bellmunt et al. [14]	Joly et al. [15]	Fechner et al. 2006 [16]	Fechner et al. 2006 [16]	Sweeney et al. [17]	Hussain et al. [18]	Wulfing et al. [19]	Hahn et al. [20]	Rosenberg et al. [21]	Gomez-Abuin et al. [22]
Chemotherapy regime	Vinflunine + Best supportive care	Best supportive care alone	Paclitaxel	Gem-Pac 3 weekly regime	Gem-Pac 2 weekly regime	Pemetrexed	Trastuzumab plus Pac, Carbo, and Gem	Lapatinib	GC + Bevacizumab	Bortezomib	Bortezomib
Response Rate (%)	8.6	0	9	50	38	27.7	70	3	67	0	0
Complete Response rate (%)	0	0	2	50	7	6.4	11.4	0	17	0	0
Median time to progressive disease (months)			3	11	6		9.3	2		1.4	1.9
Median survival (months)	6.9	4.3	7	13	9	9.6	14	4.1	19.1	5.7	3.5
Toxicity											
Grade 3 or 4 Neutropenia (%)	50	2.7		36	23	4.3	86.4		35	0	0
Neutropenic sepsis or febrile neutropenia (%)	6	0	4				40.9		2	0	0
Drug-related deaths (%)							5	1.7	7	0	0
